# A Correlational Study of Ophthalmic Artery Doppler Parameters and Maternal Blood Pressure in Normotensive and Pre-eclamptic Pregnancies at a Tertiary Care Hospital

**DOI:** 10.7759/cureus.40713

**Published:** 2023-06-20

**Authors:** Neha Kumari, Rajeev Kumar Ranjan, Nisha Rai, Anima R Xalxo, Suresh K Toppo, Paras Nath Ram

**Affiliations:** 1 Radiology, Rajendra Institute of Medical Sciences, Ranchi, IND

**Keywords:** maternal blood pressure, ophthalmic artery, normotensive, preeclampsia

## Abstract

Background

Hypertensive disorders are one of the most common complications of pregnancy. This study aimed to investigate the relationship between ophthalmic artery Doppler indices and preeclampsia development and evaluate differences in these indices between normotensive and hypertensive pregnancies.

Methods

A hospital-based cross-sectional observational study was conducted involving a sample size of 80 pregnant women: 40 normotensive and 40 preeclamptic. The participants' ophthalmic artery Doppler parameters were evaluated using ultrasonography. Various clinical and demographic factors were also collected for analysis.

Results

Significant differences in the pulsatility index (PI) and end-diastolic volume (EDV) of the ophthalmic arteries were found between the normotensive and preeclamptic participants (p < 0.05). An inverse correlation was observed between the ophthalmic artery PI (OAPI) and mean maternal arterial pressure, suggesting reduced orbital vascular resistance and increased orbital flow. Moreover, the decrease in PI was more significant in severely preeclamptic women than in mildly preeclamptic and normotensive women. The findings indicated a significant correlation between ophthalmic artery Doppler parameters and the development of preeclampsia. The decrease in OAPI was particularly profound in women with severe preeclampsia. However, the study was limited by its small sample size and the lack of matching of participants based on maternal age, gestational age, and other factors.

Conclusions

The study results suggest that ophthalmic artery Doppler parameters, mainly PI and EDV, could serve as reliable indicators for the development of preeclampsia. Given their safety, cost-effectiveness, and accessibility, these parameters can help differentiate between preeclamptic and normotensive pregnancies in late gestation. Further research with larger sample sizes and matched participant groups is recommended for more conclusive results.

## Introduction

Hypertensive disorders represent one of the prevalent pregnancy complications, affecting 7% to 15% of patients [1]. These disorders correlate with high maternal and perinatal morbidity and mortality [2]. Moreover, they are strongly linked with fetal growth restriction, low birth weight, spontaneous or iatrogenic preterm delivery in approximately 8% to 10% of cases [3], respiratory distress syndrome, admission to neonatal intensive care, and cerebral palsy [4]. The categorization of hypertensive disorders during pregnancy includes four distinct groups: gestational hypertension, preeclampsia, eclampsia, chronic hypertension (essential, secondary), and preeclampsia superimposed on chronic hypertension. In primigravidas, hypertensive disorders cause preterm delivery in 0.3% of cases (approximately one in 250) [3]. Placental disorders, such as preeclampsia, result in less than the 10th percentile of birth weight for gestation in 20% to 25% of preterm births and 13% to 19% of term births [3].

According to hemodynamic studies, generalized arteriolar vasoconstriction in preeclampsia results in hypoperfusion of the targeted organs, disruption of the blood-brain barrier, and failure of cerebral autoregulation. This failure causes cerebral vasculature, including the ophthalmic arteries, to be overperfused. The association between ophthalmic artery Doppler indices and preeclampsia does not appear to result from trophoblast invasion but may relate to maternal hemodynamic adaptation during pregnancy [5]. As a result, color Doppler has been used to visualize and measure flow in retrobulbar blood vessels [6]. Ophthalmic artery Doppler assessments performed between 35 and 37 weeks gestation can predict the subsequent onset of preeclampsia [7].

The ophthalmic artery presents an accessible opportunity to monitor maternal cardiovascular changes, particularly in hypertensive disorders of pregnancy. With functional, embryological [8], and anatomical similarities to intracranial vessels [9,10], the ophthalmic artery can provide insights into the small-caliber intracerebral vasculature and its hemodynamics that are challenging to image transcranially [9].

While magnetic resonance imaging safely allows the study of intracranial blood flow during pregnancy, it is expensive, not readily available, and contraindicated for patients with ferromagnetic implants. Other radiological imaging modalities for assessing the intracranial blood vessels, such as catheter angiography, computed tomographic angiography, and radionuclide imaging, use ionizing radiations. Hence, these modalities pose hazards to the fetus and are contraindicated in pregnancy [11]. Transcranial Doppler ultrasound is a safe and fast method to study intracranial vessels, yet it is challenging to use, offers poor spatial resolution, and requires significant technical expertise for accuracy [11]. Additionally, transcranial Doppler imaging equipment is often unavailable in many low-income nations.

Conversely, ophthalmic artery Doppler is cost-effective, accurate, repeatable, noninvasive, and objective without ionizing radiation exposure. Inspecting the ophthalmic artery is technically feasible as eyeballs lack bone, fat, or gas structures. This technique also boasts a predictive value for developing early onset preeclampsia, similar to uterine artery Doppler evaluation [5].

This study aimed to investigate potential differences in ophthalmic artery Doppler parameters between normotensive and hypertensive pregnancies and establish a correlation between ophthalmic artery pulsatility index (OAPI) and maternal blood pressure (BP).

## Materials and methods

We conducted this hospital-based cross-sectional study in the Radiology Department at Rajendra Institute of Medical Sciences (RIMS), Ranchi, Jharkhand, India, from January 2021 to November 2022. We completed a thorough literature survey over one year before we collected the sample, which fell within the study period. The Institutional Ethical Committee of RIMS provided ethical approval (Approval no. 62/IEC/RIMS, dated 17/05/2022). All participating patients gave their informed consent.

The study population included 80 pregnant women: 40 normotensive and 40 preeclamptic, all referred to the Department of Radiology for fetal well-being assessment. The sample size stemmed from a survey by Hata et al. [12]. We selected participants from various gestational periods.

Inclusion criteria for preeclamptic pregnant women required a gestational age over 20 weeks, BP equal to or above 140/90 mmHg, and proteinuria exceeding 0.3 g/l in a 24-hour urine sample or at least a 2+ on a dipstick random urine test. Severe preeclampsia required a gestational age of over 20 weeks with a sustained BP equal to or above 160/110 mmHg. We excluded women diagnosed with diabetes, those on corticosteroids, individuals with a history of addiction, ocular disease, vasculitis, or other vascular diseases, or those who did not provide consent.

We collected data, including the patient's name, age, occupation, and socio-economic status. We also took a detailed clinical history, noting presenting concerns such as headache, ankle swelling, disturbed sleep, blurred vision, or decreased urinary output. We gathered comprehensive obstetric records, noting gravida, parity, similar episodes in previous pregnancies, and medical history of hypertension or diabetes. We measured BP in the brachial artery, using the fourth Korotkoff sound to determine the diastolic pressure. We examined all pregnant women for urinary proteinuria via dipstick and 24-hour routine urine and graded them accordingly.

**Figure 1:**
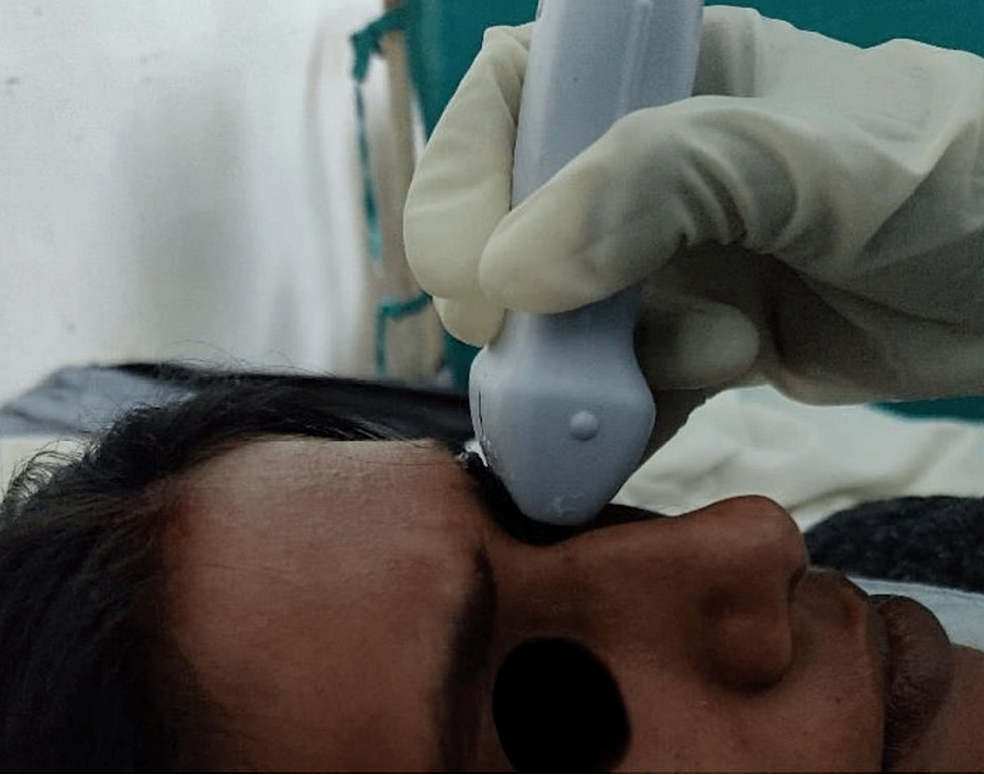
Probe position for ophthalmic artery study.

**Figure 2:**
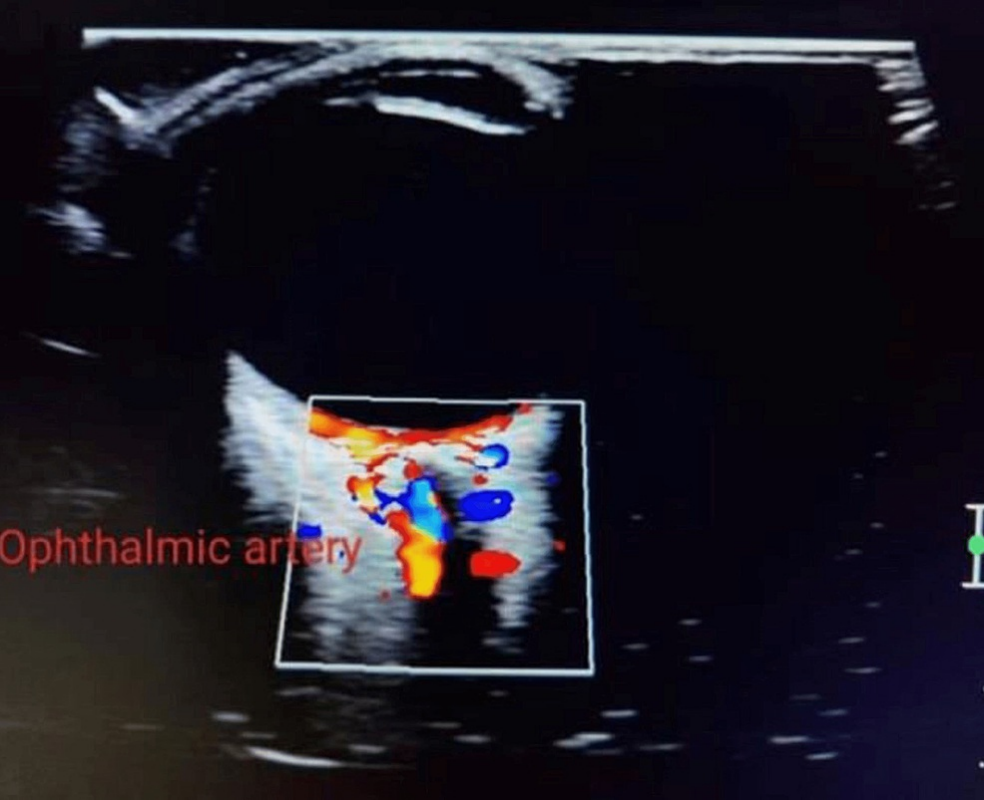
Ophthalmic artery in colour Doppler

We placed patients supine during the ultrasound examination, applying the gel directly on the closed eyelid and instructing them to keep the eyeball fixed and move as directed (Figure 1). We examined both ophthalmic arteries within the orbit, starting with the right (Figure 2). We kept the angle of insonation below 20 degrees and set the Doppler sample volume to 2 mm. We identified the ophthalmic artery on the medial side of the optic nerve using color Doppler flow imaging and measured the flow velocity approximately 15 mm from the optic disc.

**Figure 3:**
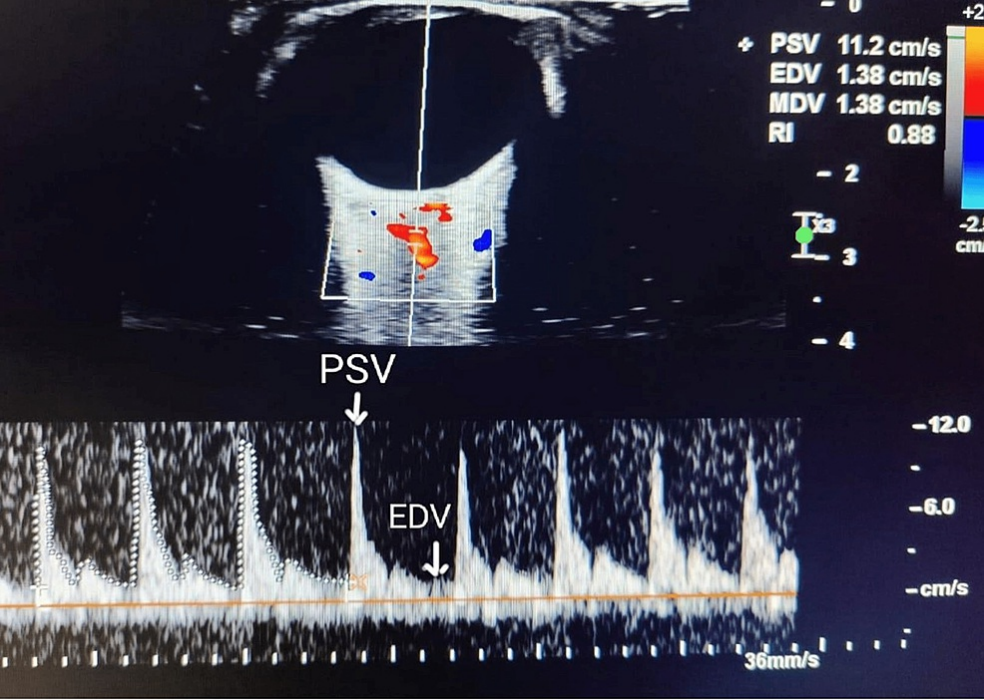
Ophthalmic artery Doppler waveform. PSV: peak systolic velocity, EDV: end diastolic velocity, MDV: mean diastolic volume, RI: resistive index

We avoided excessive eyelid compression with the transducer during the examination. We averaged the maternal ophthalmic artery peak systolic velocity (PSV), end-diastolic velocity (EDV), time-averaged mean peak velocities, and pulsatility index (PI) for each side (Figure 3). We evaluated the mean PI for statistical analysis by averaging the values obtained from the right and left sides. We measured PI, resistive index (RI), PSV, and EDV using pulsed Doppler ultrasonography with a 6- to 13-MHz linear transducer and a 1- to 5-MHz sectoral probe transducer in a Sonosite ultrasonography machine (Fujifilm Sonosite, Bothell, WA, USA). We calculated gestational age in weeks using ultrasonography per Hadlock [13] considering parameters such as crown-rump length, biparietal diameter, head circumference, abdominal circumference, and femur length. 

Data analysis

Data were entered into a spreadsheet (Excel; Microsoft Inc., Redmond, WA, USA) after coding and processed further using IBM SPSS Statistics for Windows, Version 20.0. (IBM Corp., Armonk, NY, USA). Results are presented using tables. The independent sample Student's t-test was applied to the ophthalmic arteries and Doppler parameters to analyze data among normotensive and preeclamptic pregnant women. A level of statistical significance was established, considering a p-value of less than 0.05 to be statistically significant.

## Results

We selected a sample of 80 patients for this study, comprising 50% normotensive individuals (40 patients), 35% with mild preeclampsia (28 patients), and 15% with severe preeclampsia (12 patients). Normotensive participants had a mean age of 20.8±2.9 years, ranging from 18 to 28 years, whereas the mean age of preeclamptic participants was 27.7±6.3 years, ranging from 18 to 38 years (Table 1). This difference indicates a higher age of preeclampsia onset in pregnant women.

**Table 1:** Age distribution of normotensive and hypertensive pregnant women

Maternal age (years)	Normotensive (n)	Hypertensive (n)
<20	24	6
21-30	16	18
31-40	0	16

Gestational age ranged from under 20 weeks to term in normotensive women and from 20 weeks in preeclamptic women (Table 2). Most women with preeclampsia were in their third trimester, suggesting that mild to severe preeclampsia generally occurs later in pregnancy.

**Table 2:** Distribution of patients by MAP MAP: mean arterial pressure

Group	MAP	<20 Weeks	20-28 Weeks	29 Weeks - Term	Total (n)
Normotensive	≤104 mmHg	8	18	14	40
Mild Preeclampsia	105–125 mmHg	0	4	24	28
Severe Preeclampsia	≥126 mmHg	0	2	10	12

We found statistically significant differences in the PI of the normotensive and preeclamptic participants, with mean values of 2.20 to 2.00 and 1.40, respectively (p<0.05). Similarly, the EDV in both ophthalmic arteries was statistically significant, with mean EDV values of 8.65 to 8.90 and 16.5 to 16.75 in normotensive and preeclamptic participants, respectively (p<0.05). Other Doppler parameters, such as RI, PSV, and mean velocity, showed p-values >0.05, indicating statistical insignificance (Table 3). The mean OAPI was highest in normotensive pregnant women (2.24) and lowest in the severe preeclamptic group (1.07). This inverse correlation between OAPI and mean maternal arterial pressure suggests reduced orbital vascular resistance and increased orbital flow.

**Table 3:** Ophthalmic artery velocity parameters in normotensive and preeclamptic pregnant patients. BP: blood pressure, L.PI: left ophthalmic artery pulsatility index, R.PI: right ophthalmic artery pulsatility index, LPSV: left peak systolic velocity, RPSV: right peak systolic velocity, LMNV: left mean velocity, RMNV: right mean velocity, LDV: left diastolic velocity, RDV: right diastolic velocity, SD: standard deviation, SE: standard error, CI: confidence interval * 1 = Preeclamptic, 2 = Normotensive ** P<0.05 is considered significant

Parameters	BP ^*^	Mean	SD	SE Mean	95% CI of the Difference		P-Value **
					Lower	Upper	
L.PSV (cm/s)	1	51.00	18.581	4.155	-16.898	2.598	.146
	2	58.15	10.883	2.434	-16.975	2.675	
L.EDV (cm/s)	1	16.55	5.365	1.200	4.952	10.348	.000
	2	8.90	2.594	0.580	4.918	10.382	
L.MNV (cm/s)	1	25.90	9.159	2.048	-0.683	8.683	.092
	2	21.90	4.811	1.076	-0.733	8.733	
L.PI	1	1.40	0.503	0.112	-1.094	-.506	.000
	2	2.20	0.410	0.092	-1.094	-.506	
L.RI	1	0.95	0.375	0.109	-0.368	0.268	.342
	2	1.00	0.000	0.000	-0.650	0.550	
R.PSV (cm/s)	1	54.00	15.865	3.547	-10.630	7.430	.722
	2	55.60	12.093	2.704	-10.651	7.451	
R.EDV (cm/s)	1	16.75	6.958	1.556	4.596	11.604	.000
	2	8.65	3.392	0.758	4.552	11.648	
R.MNV (cm/s)	1	26.00	9.414	2.105	-1.490	8.990	.156
	2	22.25	6.735	1.506	-1.508	9.008	
R.PI	1	1.40	0.503	0.112	-0.828	-0.372	.000
	2	2.00	0.000	0.000	-0.835	-0.365	
R.RI	1	1.05	0.224	0.050	-0.054	0.154	.336
	2	1.00	0.000	0.000	-0.055	0.155	

## Discussion

Our findings show that the mean OAPI decreases as pregnancy progresses in both normotensive and preeclamptic individuals. Hence, the OAPI is negatively correlated with gestational age. This decrease in the PI is more pronounced in severely preeclamptic women than in mildly preeclamptic and normotensive women (Table 4).

**Table 4:** Relationship between OAPI and maternal BP. OAPI: ophthalmic artery pulsatility index, BP: blood pressure, L.PI: left ophthalmic artery pulsatility index, R.PI: right ophthalmic artery pulsatility index

OAPI	Normotensive (n=40)			Mild Preeclampsia (n=28)			Severe Preeclampsia (n=12)		
	Min	Max.	Mean	Min.	Max	Mean	Min	Max.	Mean
L.PI	1.63	2.65	2.20	1.20	2.17	1.61	0.74	1.15	0.98
R.PI	1.73	2.5	2.00	1.23	2.6	1.62	1.00	1.27	1.17

We used ultrasonography to evaluate the maternal ophthalmic artery in normotensive and preeclamptic individuals. We performed color and spectral Doppler studies, collected data from both eyes and analyzed the results. We found statistically significant differences between normotensive and preeclamptic individuals in some Doppler parameters. The PI was lowest in severe preeclampsia (1.17±0.08, p<0.05) and highest in normotensive pregnant women (2.92±0.59, p<0.05). The results of the quality assessment for the included studies are shown in (Table 5).

**Table 5:** Characteristics and quality assessment of included studies EDV: end‐diastolic velocities, MV: time‐averaged mean peak velocities, NOS: Newcastle‐Ottawa Scale, NR: not reported, PI: pulsatility index, PR: peak ratio, PSV: peak systolic velocities, RI: resistance index, GA: gestational age Paper listed in the above table are from a metaanalysis by Dai X et al. [20]

Author	Year	Country	Study design	Number of participants	GA at examination	Ultrasound characteristics	Reported outcomes	NOS score
Hata et al. [12}	1995	Japan	Case control	29	>32	Patients were studied once with color Doppler flow imaging and pulsed Doppler ultrasonography after 32 weeks gestation.	PSV, MV, EDV, PI	7
Takata et al. [14]	2002	Japan	Case control	99	>32	An Aloka SSD‐2200 scanner with a 3.5‐MHz transabdominal probe (Aloka Ltd., Tokyo, Japan) was used.	PSV, MV, EDV, PI, RI, PR	8
Ayaz et al. [8]	2003	Turkey	Case control	60	>32	Ultrasound examination was performed using a 10 MHz linear transducer.	PI and RI	8
Diniz et al. [15]	2008	Brazil	Cross‐sectional	91	30.7 ± 5.0	Orbital vascular Doppler was performed using an electronic linear probe in a frequency ranging from 7–10 MHz.	PSV, EDV, PI, RI, PR	7
de Oliveira et al. [16]	2013	Brazil	cross‐sectional	379	>20	All scans were performed using Nemio (Toshiba Medical Systems Co, Ltd, Tokyo, Japan) and Sonoace X8 (Samsung Medison Co, Ltd, Seoul, Korea) high‐resolution equipment with a 7.5‐MHz linear transducer and a 50 Hz wall filter setting; the Doppler sample volume was adjusted at 2 to 3 mm.	RI, PI, PR	8
Olatunji et al. [17]	2015	Nigeria	Case control	83	NR	Transorbital triplex ultrasound scan with a 7–10 MHz multifrequency linear transducer was used.	PSV, PI EDV, RI	8
Madina et al. [18]	2020	Pakistan	Cross‐sectional	60	Second or third trimester.	The ultrasound machine used in the study (Toshiba Xerio) was equipped with a linear probe of 7–14 MHz for ophthalmic artery examination.	RI	8
Onwudiegwu et al. [19]	2020	Nigeria	Case control	143	NR	Ocular color and pulsed‐wave Doppler ultrasound examination of the ophthalmic artery was done on all participants using the LOQIC P5 GE ultrasound scanner (General Electric Healthcare, South Korea) with a 5–14 MHz linear transducer.	PI, RI, PSV, PR, EDV	7

We also found statistically significant differences in the left and right EDVs. The mean EDV was 16.65 cm/sec in preeclamptic women and 8.77 cm/sec in normotensive women (p<0.05). Previous research, such as that by Diniz et al., also found statistically significant differences in EDV (p=.001) between mild and severe preeclamptic women and healthy pregnant women [15].

Our study had a few limitations, including a small sample size, based on a survey by Hata et al. [12]. A larger sample size may have produced more precise results [21]. Additionally, the normotensive and preeclamptic women were not matched based on maternal age, gestational age, or other factors that could affect the result. Matching patients could have reduced potential unknown biases. We performed a cross-sectional study; however, a longitudinal study tracking ophthalmic artery changes from early gestation to term might have provided more temporality. Nevertheless, we opted for a cross-sectional design to avoid dropout rates that often accompany longitudinal studies.

## Conclusions

Our study found statistically significant differences in the maternal OAPI and EDV in both eyes. We observed that the OAPI inversely correlates with maternal mean arterial blood pressure and gestational age. Both variables indicate decreased vascular resistance and orbital vasodilation, similar to cerebral vessels, since the ophthalmic artery is a branch of the internal carotid artery. Therefore, these parameters can help differentiate between preeclamptic and normotensive pregnancies in late gestation. Given its safety, cost-effectiveness, and accessibility, ultrasonography can be a bedside diagnostic tool.
